# Mental health status of students during coronavirus pandemic outbreak: A cross-sectional study

**DOI:** 10.1016/j.amsu.2022.103739

**Published:** 2022-05-11

**Authors:** Seyyed Muhammad Mahdi Mahdavinoor, Mohammad Hossein Rafiei, Seyyed Hatam Mahdavinoor

**Affiliations:** a Student Research Committee, Faculty of Allied Medical Sciences, Mazandaran University of Medical Sciences, Sari, Iran; b Department of Psychology, College of Human Sciences, Shahed University, Tehran, Iran; c Student Research Committee, School of Allied Medical Sciences, Mazandaran University of Medical Sciences, Sari, Iran; d Department of Islamic Theology, Yadegar-e-Imam Khomeini (Rah) Shahre-rey Branch, Islamic Azad University, Tehran, Iran

**Keywords:** Mental health, Coronavirus, Anxiety, Stress, Depression

## Abstract

**Introduction:**

University students have been extensively affected with psychological problems due to outbreak of COVID-19 pandemic given their special position and status. In this study, we intend to examine the prevalence of depression, anxiety and stress among a group of students.

**Methods:**

This cross-sectional study was performed in medical Sciences universities of Mazandaran Province. 352 students were recruited by simple available sampling method. Data collection tools were Standard Mental Health Questionnaire (DASS-21) and demographic information questionnaire. Data analysis was done using SPSS software through descriptive and analytical statistics.

**Results:**

The mean age of students was 22.44 ± 3.4 and 54.3% of them were females. 33.6, 28.4, and 27.3% of students had moderate to extremely severe levels of symptoms of depression, anxiety, and stress, respectively. There was a significant relationship between total mental health score with physical activity (p < 0.04) and with smoking (p < 0.02). There was also a significant relationship between smoking and depression (p < 0.01).

**Conclusion:**

Considering the fact that anxiety, stress and depression are common among medical sciences students of Mazandaran Province, necessary measures must be taken to improve their mental health status.

## Highlights

• Considering the fact that anxiety, stress and depression are common among medical sciences students of Mazandaran Province.
• A significant correlation was observed between total DASS-21 score with the amount of physical activity and with smoking.
• There was also a significant correlation between smoking and depression (p < 0.01).

## 1 Introduction

For the first time in late December 2019, cases of pneumonia of unknown cause were reported in Wuhan, China, and later in January 2020, a new strain of coronavirus was identified as the causative agent of these pneumonia cases. World Health Organization (WHO) has chosen the official name COVID-19 (abbreviation for Corona 2019) for this disease. On March 11, 2020, WHO declared COVID-19 as a pandemic disease. The virus quickly spread to all countries of the world and caused outbreaks in most countries [1,2]. COVID-19 has initiated a global health crisis since its outbreak in Wuhan, China, and it is a Public Health Emergency of International Concern (PHEIC) that not only threatens people's lives but also their mental health [3]. In order to decelerate the spread of the disease and protect the health systems from excessive pressure, many countries imposed restrictions on population movement as well as full or partial quarantine, including restrictions on leaving the house, travel and visitation limits and so on [4]. Because these critical conditions (i.e. epidemics and quarantine) are harmful by themselves, painful emotions are part of their consequences for human life [5]. People were not psychologically prepared for the epidemic because of the sudden nature, severity and negativity of the emergency [3]. Fear of illness combined with quarantine and physical alienation may lead to social isolation, loss of income, loneliness, inactivity, limited access to basic services, further exposure to food, alcohol, online gambling, and decreased family and community support, especially for the elderly and vulnerable people [4,6]. The researchers found that this pandemic has led to mental health problems such as depression and anxiety [7,8]. COVID-19 pandemic is now an urgent issue in global mental health and an unprecedented challenge for the health systems of all countries, and the destructive effects of this pandemic on mental health become more apparent over time [7,8]. Some people are more exposed to the psychological problems caused by this pandemic than the general population due to their special situation, including students and health care professionals [3,9]. Various studies report that students' mental health has deteriorated during this outbreak [3,[10], [11], [12], [13]].

Admission to university causes a change in social, family and individual life of students and is therefore a highly sensitive period. Students are vulnerable to depression, anxiety and stress due to factors such as curriculum structure, the need to meet academic requirements, frequent exams, high functional stress, fear of failure, learning environment, lack of time to take care of themselves and communicate with family and friends [14,15]. In addition to the fact that entering university and changing the environment and lifestyle are stressful for students by themselves, the outbreak of COVID-19 has made the situation more difficult for students and has psychologically damaged them. Factors such as changing educational environment from face to face to online, physical and social isolation, disruption of daily routines, financial stress, inactivity, spread of corona-related rumors, fear of getting infected by COVID-19, death of loved ones, and so forth double the psychological stress to students [3,4,6,8,13]. In Iran, the situation went even further deteriorated. Economic sanctions, lack of resources, and poor governance led to shortage of hospital beds, drugs such as tocilizumab and even intravenous fluids. Iranian supreme leader, Ali Khamenei, banned the import of American and British vaccines because of his pessimism about the West, and such sources of vaccine supply were severely limited. While by January 21, 2021, more than 7% of the world's population had been fully vaccinated, vaccination rate in Iran was only 1.1%. The percentage of positive tests sometimes reached more than 30%, which indicates the lack of diagnostic kits. Also, due to the mismanagement of the then government and economic sanctions, the livelihood packages were not sent to people's houses, so the government could not fully implement the quarantine. Due to the above reasons, the critical situation due to COVID-19 outbreak was prolonged. While many countries were in the second wave or in the worst case of the third wave, Iran had passed the fourth wave of the epidemic and was waiting for the fifth wave [[16], [17], [18]]. Studies have shown that the risk of mental health problems increases in long-term catastrophic environments. In Iran, the prevalence of psychological problems among students gradually increased due to the prolongation of the crisis [3,19]. In a study conducted by Ghafari et al. during the COVID-19 pandemic in an Iranian university, it was found that 11.4%, 16.1% and 27.5% of students suffer from suicidal ideation, severe depression, and high levels of anxiety, respectively [19]. Given the importance of students' mental health, especially during the outbreak of COVID-19, there is an urgent need to assess and monitor the unprecedented burden of mental health on them [14,20], which could prevent the consequences of mental health by addressing the root cause of the problem and implementing effective prevention programs [20].

In this study, we intend to measure the prevalence of anxiety, stress and depression in medical sciences students in Mazandaran Province during the outbreak of COVID-19.

## 2 Materials and methods

### 2.1 Research design

This descriptive-analytical study was conducted from May 30 to June 18, 2021 in medical sciences universities of Mazandaran Province located in the north of Iran. Due to its tourist attractions, this province is one of the most populous tourist destinations in Iran. There are two medical sciences in Mazandaran Province. The first is Mazandaran University of Medical Sciences, which is located in Sari city, the capital of Mazandaran Province. Another is Babol University of Medical Sciences, which is located in Babol city. Most faculty members and staff of these two universities are native to Mazandaran. In 2021, both universities were among first grade universities in the ranking of Ministry of Health and Medical Education of Iran, so the quality of education is high in both universities. Tuition is free in both universities, which increases competition for admission. Given the similarities between the two universities and the fact that both of them are in the same social and cultural zone, we decided to choose samples from both universities.

### 2.2 Sample and sampling method

The statistical population of this study included all students who were studying at Mazandaran University of Medical Sciences and Babol University of Medical Sciences at the time of filling out the questionnaire. G-POWER software (version 3.1.9.2) was used to determine the sample size. Taking into account the probability of first type (alpha) error at 0.05 level (95% confidence), the acceptable level of test power equal to 0.95 and the effect size of 0.2, the sample size was estimated to be 327. Therefore, the sample size (N = 352) was good for the main purpose of this study. Samples were collected by convenience sampling method. Three hundred and sixty-six students who wished to participate in this study filled out the questionnaires. Fourteen students who filled out the questionnaires were from other universities, so we excluded them from the study before starting the analysis. Finally, 352 questionnaires remained that were subject to the analysis process.

Inclusion criteria were as follows: being a student of Mazandaran University of Medical Sciences and Babol University of Medical Sciences and willingness to participate in this study.

Exclusion criteria were students not studying at Mazandaran University of Medical Sciences and Babol University of Medical Sciences.

### 2.3 Measurement instrument

The data collection tool was a two-part questionnaire. The first part was devoted to examining the personal characteristics of the samples (gender, age, marital status, field of study, degree, name of university, admission year, family economic status, province of residence, amount of physical activity, smoking, history of COVID-19) with 12 items. The second part was the short form of Depression, Anxiety and Stress scale (DASS-21) introduced by Lovibond and Lovibond [21,22]. This is a self-report questionnaire that consists of 21 items and 3 subscales of anxiety, stress and depression, each with 7 items. The depression subscale assesses inadequacy, dissatisfaction, hopelessness, devaluation, and inertia. Anxiety subscale measures acute responses to fear as well as physical and mental symptoms of anxiety, and stress subscale evaluates tension, restlessness, irritability, and difficulty relaxing [23]. The validity and reliability of this scale in Iran were measured in different studies and for different groups, which was acceptable [23,24]. The answer to this scale is in the form of a 4-point Likert scale (Did not apply to me at all; Applied to me to some degree, or some of the time; Applied to me to a considerable degree, or a good part of time; Applied to me very much, or most of the time).

### 2.4 Data analysis

We analyzed data using Statistical Package for Social Sciences (SPSS v.22.0; SPSS Inc., Chicago, IL, USA) by descriptive (frequency, mean, standard deviation) statistics. After examining the normal distribution of data by Kolmogorov-Smirnov test, we used Mann-Whitney, Kruskal Wallis and Chi-square tests to determine the correlation between demographic variables and mental health status. P < 0.05 was considered as the significance level.

### 2.5 Ethical consideration

This study was approved by Research Council of Mazandaran University of Medical Sciences as well as by ethics committee of that university with ethics code IR.MAZUMS.REC.1400.334.

In this study, students were completely free to participate in the study and were not asked to show any signs indicating their identity.

## 3 Results

**Fig. 1:**
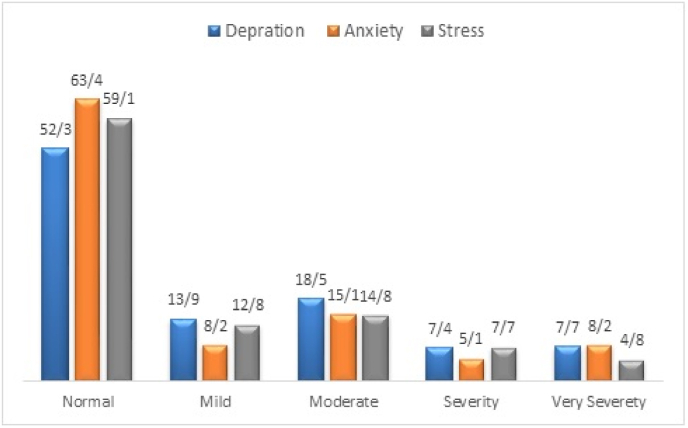
Status of depression, anxiety and stress subscales of students' mental health.

A total of 352 participants completed the questionnaires. The age range of students was 18–45 years with mean age of 22.44 ± 3.4, and 54.3% of them were female. There were 30.7% physicians, 33.5% paramedics, 11.9% nurses, and the rest were from various fields of medicine, pharmacy, health and New Medical Technologies. A history of COVID-19 was reported by 32.7% of students. Other demographic information is given in Table 1. The mean mental health status of students based on DASS-21 was 36.93 ± 11.99 out of a total score of 126 (CI95% = 35.7–38.9). Of the total students, 33.6, 28.4, and 27.3% had moderate to extremely severe symptoms of depression, anxiety, and stress, respectively (Fig. 1). There was a significant correlation between the total score of DASS-21 and amount of physical activity (p < 0.04) and smoking (p < 0.02). There was also a significant correlation between smoking and depression (p < 0.01).

**Table 1:** Socio-demographic characteristic of students and their relationship with mental health subscales during COVID-19 epidemic (n = 352).

Variables		N (%)/Mean ± SD	DASS 21	P-Value	Deration	Anxiety	Stress
					P-Value***	P-Value***	P-Value***
Sex	Male	191(54.3)	32.73 ± 24.3	0.48*	0.10	0.37	0.33
	Female	161(45.7)	30.87 ± 23.6				
Age	–	22.44 ± 3.4	36.93 ± 11.9	0.61	0.96	0.24	0.82
Marital status	Single	330(93.8)	31.58 ± 24.0	0.26*	0.45	0.71	0.98
	Married	22(6.3)	36.18 ± 23.1				
Faculties	Medicine	108(30.7)	33.82 ± 23.1	0.12**	0.18	0.23	0.21
	Dentistry	32(9.7)	31.00 ± 21.2				
	Pharmacy	28(8)	23.70 ± 17.2				
	Health	18(5.1)	30.44 ± 24.6				
	Nursing	42(11.9)	23.38 ± 16.1				
	Paramedicine	118(33.5)	35.52 ± 27.4				
	Medical tech	3(0.9)	22.66 ± 15.2				
Degree	Undergraduate	169(48.0)	32.00 ± 25.6	0.54**	0.68	0.47	0.17
	Postgraduate	179(50.8)	31.76 ± 22.3				
Year level	First year	64(18.2)	33.74 ± 25.8	0.77**	0.70	0.58	0.71
	Second year	89(25.3)	32.41 ± 25.1				
	Third year	60(17)	27.18 ± 20.4				
	Fourth year	47(13.4)	34.32 ± 27.1				
	Fifth year	23(6.5)	28.18 ± 19.0				
	Sixth year	14(4)	33.55 ± 22.1				
	Higher education	7(2)	32.00 ± 16.4				
Region	1	248(70.5)	30.10 ± 22.7	0.40**	0.73	0.73	0.53
	2	12(3.4)	35.16 ± 25.2				
	3	27(7.7)	37.55 ± 26.1				
	4	7(2)	46.85 ± 40.5				
	5	48(13.6)	37.16 ± 28.7				
Financial level	Low	30(8.5)	26.33 ± 21.9	0.31**	0.84	0.55	0.18
	Moderate	298(84.7)	32.34 ± 24.2				
	Good	23(6.5)	32.78 ± 23.5				
Physical activity	Less than 3 h	223(63.4)	34.10 ± 24.1	0.20**	0.46	0.30	0.54
	3 - 6	82(23.3)	26.60 ± 22.0				
	More than 6 h	46(13.3)	30.60 ± 25.5				
Smoking	No	294(83.5)	32.26 ± 24.1	0.02**	**0.01**	0.14	0.16
	Yes some times	44(12.5)	25.59 ± 21.3				
	Yes every day	14(4)	43.57 ± 25.4				
Covid-19	No	237(67.3)	32.32 ± 22.9	0.23*	0.51	0.15	0.39
	yes	115(32.7)	30.95 ± 26.0				

* Mann-Whitney U - **Kruskal Wallis - *** Chi-square.

The Iranian Ministry of Interior has grouped the provinces of Iran into five regions according to their regional and cultural commonalities [25]. In this study, we also divided the students' accommodation according to the grouping of Iranian Ministry of Interior. The results showed that none of DASS-21 subscales and total score of DASS-21 were related to students' place of residence. Other demographic characteristics were not related to total scores and subscales of depression, anxiety and stress (Table 1).

## 4 Discussion

The aim of this study was to investigate the prevalence of anxiety, stress and depression among students of medical sciences universities in Mazandaran Province during coronavirus outbreak. According to our results, about one and a half years after the onset of COVID-19, 33.6%, 28.4%, 27.3% of students had moderate to extremely severe levels of depressive, anxiety and stress symptoms, respectively, which is significantly different from that beforehand [26]. Studies show that the risk of psychological problems due to epidemic increases in long-term catastrophic environments [3]. LI et al. in a study showed that the mental health of students is gradually deteriorating since the outbreak of COVID-19 pandemic [3]. In Iran, the prevalence of psychological problems among students progressively increased due to the prolongation of the crisis [19]. In a study by Nakhostin-Ansari et al. [27], two months after confirmation of the first case of COVID-19 in Iran, it was found that 14% of medical students at Tehran University of Medical Sciences had moderate to severe anxiety and that 11.8% had moderate to severe depression. Also, in the study by Ghafari et al., about 9 months after COVID-19 arrived in Iran, it was found that 11.4% of Tabriz University of Medical Sciences students suffer from suicidal ideation, 16.1% from severe depression and 27.5% from high levels of anxiety [19]. In a systematic review and meta-analysis by Li et al. [3], the prevalence of depression and anxiety among students worldwide was estimated 39% and 36%, respectively, which was significantly lower than the results of this study (Depression: 47.7% and Anxiety: 36.6%)(Fig. 1). Given the crisis that Iran has had for years, this difference is justifiable. Sanctions, mismanagement and corruption have increased inflation rate and spread poverty, unemployment, class divisions, shortages of medicine and medical equipment, and so on [16,28,29]. According to studies, the mental health of Iranians was worsening before the outbreak of COVID-19 pandemic, and the disease only aggravated it [30,31].

In the results of our study, a significant correlation was observed between total DASS-21 score with the amount of physical activity (p < 0.04) and with smoking (p < 0.02). There was also a significant correlation between smoking and depression (p < 0.01). According to previous studies, the amount of physical activity and mental health status are positively related [32,33]. However, in a study by Kim et al., a sigmoid relationship was found between physical activity and mental health status. In a sample of 7774 people from the United States, the optimal threshold for mental health benefits was found to be between 2.5 and 7.5 h of physical activity per week [34]. In this study, we found a sigmoid relationship between physical activity and mental health status. However, given the different types of question inquired from students, the optimal threshold for mental health benefits was estimated to be between 3 and 6 h of physical activity per week. Students who exercise more than 6 h a week are likely to do so professionally and for championship tournaments and hence may be subject to other stresses.

The relationship between smoking and mental health status was also sigmoid; in other words, people who smoked occasionally had better mental health than those who smoked regularly or did not smoke at all.

Other demographic information (age, gender, marital status, field of study, degree, year of study, area of residence, economic status of family and history of COVID-19) had no significant relationship with anxiety, stress, depression and overall DASS-21 score. Of course, different studies have shown various results. The relationship between demographic variables and mental health can be due to cultural differences in the target community. For example, in some studies, women have significantly higher levels of anxiety, stress and depression than men [35], but in our study, they were not significantly different with men. Given that our target population was medical sciences students, health-related education may have eliminated gender differences in personal perception of risk, and therefore there was no significant difference between men and women in mental health status [36].

Throughout history, many infectious diseases have become widespread. COVID-19 pandemic was the first outbreak of infectious disease in 21st century, but it will not be the last [31]. Previous studies have indicated that not only COVID-19 pandemic but also the overall prevalence of infectious diseases such as influenza or SARS has a negative effect on mental health status [8,[37], [38], [39]]. Therefore, governments should not only pay attention to the mental health of different groups of people during the outbreak of COVID-19 but should also have a long-term plan to reduce the effects of future epidemics and pandemics on human mental health. One of the things governments can do is planning to help people find their meaning in life. As Viktor Frankl, the founder of logotherapy says, people who have meaning and purpose in life can endure difficult situations more easily [40]. Having meaning in life helps reduce anxiety as well as decreasing the overreaction of autonomic nervous system to emotional stress [41], which is inversely related to risk factors such as substance abuse [42,43]. In other words, a person who finds meaning in life can endure suffering and sorrow and survive difficult and extremely problematic conditions of life [44]. Thus, the concept of meaning of life may be the roadmap to overcoming a mental health crisis in difficult conditions such as pandemics, and it can probably play a vital role in this regard. Logotherapy can have a positive effect on the meaning and quality of life to prevent nihilism [45,46].

### Limitation

The students' mental health status was the focus of this research so only depression, anxiety and stress were covered in this study. It is better to investigate the causes of students' mental problems in future research.

## Ethical approval

This study was approved by Research Council of Mazandaran University of Medical Sciences as well as by ethics committee of that university with ethics code IR.MAZUMS.REC.1400.334.

## Sources of funding

There is no funding support to report.

## Author contribution

SMMMN conceived the idea, designed the study and, collected the data. MHR did statistical analysis. The final report and articlewere written by SMMMN and SHM. All authors reviewed and approved the final manuscript.

## Registration of research studies

Name of the registry: researchregistry.com.

Unique Identifying number or registration ID: researchregistry7653.

Hyperlink to your specific registration (must be publicly accessible and will be checked): https://www.researchregistry.com/browse-the-registry#home/registrationdetails/620df07892096c001eaf8758/

## Guarantor

Seyyed Muhammad Mahdi Mahdavinoor.

## Provenance and peer review

Not commissioned, externally peer reviewed.

## Declaration of competing interest

SHMN and SMMMN are members of a family.
